# MicroRNA-155 contributes to enhanced resistance to apoptosis in monocytes from patients with rheumatoid arthritis

**DOI:** 10.1016/j.jaut.2017.01.002

**Published:** 2017-05

**Authors:** Megha Rajasekhar, Anton M. Olsson, Kathryn J.A. Steel, Mirella Georgouli, Ushan Ranasinghe, Christine Brender Read, Klaus S. Frederiksen, Leonie S. Taams

**Affiliations:** a Centre for Inflammation Biology and Cancer Immunology, Division of Immunology, Infection and Inflammatory Disease, King's College London, United Kingdom; b Novo Nordisk A/S, Novo Nordisk Park, DK-2760 Måløv, Denmark

**Keywords:** Synovial fluid, Monocyte, Mir-155, Cell death, MicroRNAs, Microarray

## Abstract

Monocytes and macrophages are key mediators of inflammation in rheumatoid arthritis (RA). Their persistence at the inflammatory site is likely to contribute to immunopathology. We sought to characterise one mechanism by which persistence may be achieved: resistance to apoptosis and the role of mir-155 in this process. CD14+ monocytes from peripheral blood (PBM) and synovial fluid (SFM) of RA patients were found to be resistant to spontaneous apoptosis relative to PBM from healthy control (HC) individuals. RA SFM were also resistant to anti-Fas-mediated apoptosis and displayed a gene expression profile distinct from HC and RA PBM populations. Gene expression profiling analysis revealed that the differentially expressed genes in RA SFM vs. PBM were enriched for apoptosis-related genes and showed increased expression of the mir-155 precursor BIC. Following identification of potential mir-155 target transcripts by bioinformatic methods, we show increased levels of mature mir-155 expression in RA PBM and SFM vs. HC PBM and a corresponding decrease in SFM of two predicted mir-155-target mRNAs, apoptosis mediators CASP10 and APAF1. Using miR mimics, we demonstrate that mir-155 over-expression in healthy CD14+ cells conferred resistance to spontaneous apoptosis, but not Fas-induced death in these cells, and resulted in increased production of cytokines and chemokines. Collectively our data indicate that CD14+ cells from patients with RA show enhanced resistance to apoptosis, and suggest that an increase in mir-155 may partially contribute to this phenotype.

## Highlights

• CD14+ cells from the inflamed RA joint are strongly resistant to death.
• Microarrays show differences in apoptosis genes in CD14+ cells from the RA joint.
• Mir-155 is increased and its targets decreased in RA joint CD14+ cells.
• Overexpression of mir-155 increases apoptosis resistance of healthy CD14+ cells.

## 1 Introduction

Monocytes and macrophages are key players in rheumatoid arthritis (RA) (reviewed in Ref. [1]). CD14+ monocytes from the peripheral blood (PBM) and CD14+ cells from the synovial fluid of patients with RA (likely consisting of monocytes and macrophages and henceforth referred to as SFM) have an activated phenotype [2], [3], [4], [5] and produce pro-inflammatory cytokines such as IL-1β and TNF-α [5], [6], [7]. SFM can differentiate to osteoclasts that mediate bone erosion [8], [9], [10] and have also been shown to promote production of the osteoclastogenic cytokine IL-17 by CD4+ T cells [2], [4]. The importance of the role of macrophages in RA is further supported by the observation that the degree of joint destruction correlates with synovial macrophage infiltration [11] and that successful therapy is associated with a decrease in synovial lining macrophages [12].

Studies in mice have shown that defects in monocyte apoptosis result in enhanced systemic inflammatory arthritis [13]. Various death receptors are important in mediating monocyte/macrophage apoptosis including Fas, TRAIL-R and TNF-R1. Binding of these receptors leads to recruitment of intracellular adaptor proteins, which in turn activate caspase-8/10. This forms the extrinsic apoptosis pathway. In contrast, the intrinsic apoptosis pathway, which results from intracellular stresses, leads to mitochondrial permeabilisation and formation of the ‘apoptosome’ consisting of cytochrome c, Apaf1 and caspase-9. Both pathways lead to downstream caspase cascade activation resulting in cell death (reviewed in Refs. [14], [15]).

It has previously been shown that SFM from patients with RA are resistant to apoptosis mediated by an agonistic anti-Fas antibody [16] and have increased expression of the intracellular anti-apoptotic proteins Mcl-1 and FLIP [16], [17]. Previous work from our laboratory has shown that RA SFM are also resistant to Fas-mediated killing by CD4+CD25− responder T cells, which can express FasL and induce Fas in CD14+ monocytes [18]. In addition, PBM from RA and Juvenile Idiopathic Arthritis (JIA) patients are resistant to spontaneous apoptosis [19], [20]. This resistance to cell death may lead to persistence of inflammatory monocytes and/or macrophages thereby perpetuating joint inflammation.

MicroRNAs (miRNAs) are genomically encoded small RNA molecules that are present in all multi-cellular organisms and are involved in a wide range of developmental and cellular processes. Following sequential cleavage of a long primary RNA transcript, the 19–22 bp mature miRNA is incorporated into the RNA-induced silencing complex (RISC). The miRNA then binds to target sites in 3′ untranslated regions of messenger RNA (mRNA) while the protein RISC complex inhibits translation of the mRNA (reviewed in Ref. [21]). Each miRNA is bioinformatically predicted to target hundreds of mRNA molecules. Although the degree of repression observed for each mRNA is usually small, miRNAs are believed to impact on specific phenotypes by targeting multiple mRNAs within a pathway or network of genes [22].

MiRNAs are influential mediators of monocyte-driven inflammation. In human monocytes mir-146a/b, mir-155 and mir-9 are up-regulated in response to TLR stimulation and regulate targets such as TRAF6, SHIP-1 and NFKB [23], [24], [25]. While miRNAs have been studied in detail in collagen-induced arthritis (CIA) in mice, fewer reports exist on miRNAs in RA. The expression of miRNAs has been examined in RA peripheral blood mononuclear cells [26], [27], CD4+ T cells [28], [29], fibroblast-like synoviocytes [27], [30] and synovial tissue [30]. Kurowska-Stolarska et al. reported increased expression of mir-155 in RA SFM compared to PBM and increased pro-inflammatory cytokine production as consequence [31]. The study also confirmed the immune suppressor SHIP-1 as a target of this miR.

As mir-155 has been reported to be upregulated in inflammation in general and RA SFM in particular, we sought to investigate whether there is a direct role for this miRNA in the resistance to apoptosis of RA SFM. In this report we confirm the finding that mir-155 is upregulated in RA SFM compared to RA and HC PBM, and present data identifying mir-155 as a regulator of monocyte/macrophage apoptosis.

## 2 Materials and methods

### 2.1 Patients and healthy donors

Healthy control individuals were recruited from staff and students of King's College London and Guy's Hospital, London. RA and PsA patients were recruited from the Guy's and St. Thomas' NHS Foundation Trust Rheumatology out-patient clinics and consented for donation of PB and where available SF. RA patients fulfilled the 2010 ACR/EULAR criteria whilst PsA patients fulfilled the classification criteria of the Classification of Psoriatic Arthritis (CASPAR) Study Group. All patients had an examination of tender and swollen joints and disease activity score of 28 joints (DAS28) recorded. Laboratory investigations included C-reactive protein (CRP) and erythrocyte sedimentation rate (ESR). Demographic and clinical data are shown in Supplementary Table I. All subjects provided written informed consent, and ethical approval was granted by the Bromley Research Ethics Committee.

### 2.2 Cell isolation

Mononuclear cells were isolated from PB and SF by density gradient centrifugation using Lymphoprep (PAA Laboratories/GE Healthcare Life Sciences, Buckinghamshire, UK). CD14+ monocytes were isolated from PBMC/SFMC using a positive selection kit (Miltenyi Biotec, Gladbach, Germany) (purity >95%) or by FACS sorting (purity >98%) on a BD AriaII following staining with an anti-CD14-APC-Cy7 antibody (Clone HCD14; Biolegend, San Diego, CA, USA).

### 2.3 Apoptosis assays

To assess spontaneous apoptosis, MACS isolated CD14+ cells were plated in a 48-well plate (0.5 × 10^6^/well) in a final volume of 500 μL/well of complete medium (RPMI 1640, 10% FCS, 1 × Pen/Step (Life Technologies, Carlsbad, CA, USA) and l-glutamine (2 mM, Life Technologies)) and incubated at 37 °C (5% CO_2_). Apoptosis was assessed by Annexin/7-AAD staining 17–22 h later. For anti-Fas antibody mediated apoptosis, freshly isolated or mimic transfected cells were incubated as above with the addition of agonistic anti-Fas antibody (clone CH11, Merck Millipore, Hertfordshire, UK) or IgM isotype control (Biolegend) at 50 ng/mL or 200 ng/mL for 17–22 h, followed by Annexin/7-AAD staining. Staining for Annexin and 7-AAD was performed using an ‘Apoptosis Detection Kit’ (Biolegend or Becton Dickinson). Briefly, following overnight culture the plate was placed on ice for 15 min and the cells collected, washed once in FACS buffer (PBS, 0.5% BSA, 0.1% NaN_3_) and resuspended in the supplied Annexin buffer (50 μL). Each sample was stained with 0.25 μL Annexin-FITC and 1 μL 7-AAD solutions and incubated at room temperature, in the dark for 15 min. The cells were then diluted in Annexin buffer up to 250 μL and fluorescence acquired on a BD FACS Canto within 30 min.

### 2.4 Microarrays

Total RNA was extracted from isolated CD14+ cells using TRIzol reagent (Life Technologies) and the aqueous phase further purified using RNeasy MinElute Cleanup Kit (Qiagen). RNA integrity was confirmed on an Agilent 2100 Bioanalyzer using total RNA nano chips (Agilent Technologies, Santa Clara, CA, USA). A sample was deemed to be of sufficient quality if it had an RNA Integrity Number (RIN) ≥ 6 paired with a visual inspection of the profile. Total RNA (100 ng) was used to prepare targets by 3′ IVT Express kit (Affymetrix, Santa Clara CA, USA) following manufacturer's instructions, and cocktails hybridized onto Human Genome U133 plus 2 Arrays (Affymetrix). Chips were scanned and gene expression data were normalized using the RMA algorithm and the Bioconductor package “Affy” (http://www.bioconductor.org). A custom chip definition file from brainarray.mbni.med.umich.edu was used for data extraction and analysis. Data analysis was performed using Qlucore Omics Explorer 3.0 software (Qlucore AB, Lund, Sweden). The microarray data are deposited in Gene Expression Omnibus (GEO) with accession number GSE71370.

### 2.5 qRT-PCR

Total RNA was extracted using TriZol reagent (Life Technologies) and the miRNeasy kit (Qiagen, Hilden, Germany). Mature miRNA levels were quantified using MicroRNA RT kit and TaqMan miRNA assays (Life Technologies) for mir-155 and the housekeeping RNAs RNU44 and RNU48, following manufacturer's protocols. For amplification of mRNA molecules, TriZol extracted RNA was reverse transcribed using a High Capacity cDNA Reverse Transcription Kit (Life Technologies) and amplified using SensiFAST SYBR Green Master mix (Bioline, London, UK) on an ABI7900HT machine.

### 2.6 Transfection

Transfection of CD14+ cells was performed using the NTER nanoparticle transfection reagent (Sigma, St Louis, MO, USA) following manufacturer's instructions. The cells were plated (0.5 × 10^6^/well, 48-well plate), followed by addition of medium and the transfection mix to a final volume of 250 μL, and thorough pipette mixing prior to incubation. Transfection efficiency was confirmed by flow cytometry using a scrambled control mimic conjugated to the fluorescent dye Dy547 (GE Dharmacon, Lafayette, CO, USA). MiRIDIAN miRNA mimics (final concentration 20 nM) were used for over-expression of miRNA, with a negative control from the same manufacturer (GE Dharmacon). Apoptosis was assessed 40 h after transfection.

### 2.7 Luminex

Culture supernatants were collected at the end of the culture period and immediately frozen at −80 °C. The samples were thawed and secreted cytokines were measured using a Cytokine Human 25-plex panel (Life Technologies) on a Luminex platform following manufacturer's instructions.

### 2.8 Statistical analysis

Data were tested for normality using the D'Agostino and Pearson omnibus normality test where possible. Significance testing was performed using GraphPad Prism 5 software (GraphPad, San Diego CA, USA). Tests for parametric data were applied where indicated and where t-tests were used, Welch's correction was routinely used as some data showed unequal variances. When data were not normally distributed or when biological replicates were less than 8, non-parameteric tests were used. One-way ANOVAs were performed with using Kruskal-Wallis with Dunn's post test.

## 3 Results

### 3.1 CD14+ cells from patients with RA show enhanced resistance to apoptosis

**Fig. 1:**
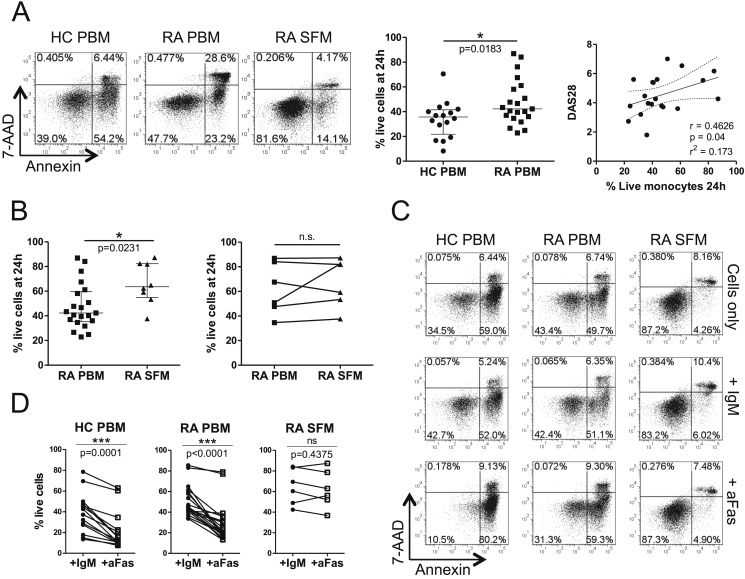
**CD14+ monocytes from patients with RA show enhanced resistance to spontaneous and Fas mediated death**. (A) MACS isolated CD14+ cells from the peripheral blood (PBM) of RA patients and healthy controls (HC) and synovial fluid (SFM) of RA patients were cultured overnight in complete medium and their survival assessed by Annexin/7-AAD staining, with the double negative population considered ‘live’. Representative data are shown in the left panel and cumulative data for HC vs. RA PBM in the middle panel (unpaired *t*-test with Welch's correction), with the correlation (Spearman's R) of spontaneous RA PBM survival at 24 h with patients' DAS28 scores in the right panel. Lines show linear regression and 95% confidence intervals. (B) Cumulative CD14+ cell survival in unpaired (left panel, unpaired *t*-test with Welch's correction) and paired (right panel, Wilcoxon matched pairs signed rank test) RA PBM and SFM. (C, D) PBM and SFM were cultured overnight with medium alone (Cells only) or with an agonistic anti-Fas antibody (aFas) or the isotype control (IgM) at 200 ng/mL and the percentage of live cells assessed. Representative dotplots (C) and cumulative data (D) are shown (Wilcoxon matched pairs signed rank test). *p < 0.05, **p < 0.01, ***p < 0.001.

Previous work from our laboratory has reported the resistance of RA PBM and SFM to Fas-mediated killing by responder T-cells [18]. To assess if these cells were also resistant to spontaneous death, CD14+ cells from the PB of healthy controls (HC) and patients with RA, and from SF from RA patients were plated in complete medium overnight and their survival quantified by Annexin/7-AAD staining. An increase in the percentage of live RA PBM was observed compared to HC PBM (Fig. 1A). A similar observation was made in age-matched samples (Supplementary Fig. 1). The percentage of live PB-derived monocytes was found to correlate with the patients' Disease Activity Score (DAS28) and CRP but not with ESR or disease duration (Fig. 1A and Supplementary Fig. 2). When we examined RA SFM, a greater percentage of live cells was detected after overnight culture compared with RA PBM (Fig. 1A and B, left panels). The increased survival of SF-derived monocytes appeared less prominent when paired RA PBM/SFM samples were compared (n = 6, Fig. 1B, right panel), however this may be explained by an enhanced apoptosis resistance in the PBM of these patients (average percentage live monocytes 62% in PBM from paired samples as compared to an overall average of 48%).

We also tested whether CD14+ cells from HC and RA PBM and RA SFM were resistant to killing by an agonistic anti-Fas antibody (clone CH-11). While HC and RA PBM were equally susceptible to killing by the anti-Fas antibody relative to isotype control treated cells, RA SFM were more resistant (Fig. 1C and D). Similar results were obtained when paired RA PBM/SFM were compared (n = 4, data not shown) or when a lower concentration of anti-Fas Ab was used (50 ng/ml, data not shown). Together these data show that CD14+ cells from RA patients, particularly those from the inflamed joint, are more resistant to cell death, relative to HC CD14+ cells. Although both RA PBM and RA SFM showed an increased resistance to spontaneous cell death, relative to HC PBM, only SFM showed enhanced resistance to Fas-induced death.

To assess whether this resistance to apoptosis was specific to RA or a more general feature of arthritis, we tested a small number of PBM and SFM from patients with psoriatic arthritis (PsA). While there was no clear difference in spontaneous or Fas-induced apoptosis between HC PBM and PsA PBM, PsA SFM tended to be more resistant to both spontaneous and Fas-mediated apoptosis (Supplementary Fig. 3).

### 3.2 Gene expression profiling shows changes in apoptosis related genes in RA SFM vs PBM

**Fig. 2:**
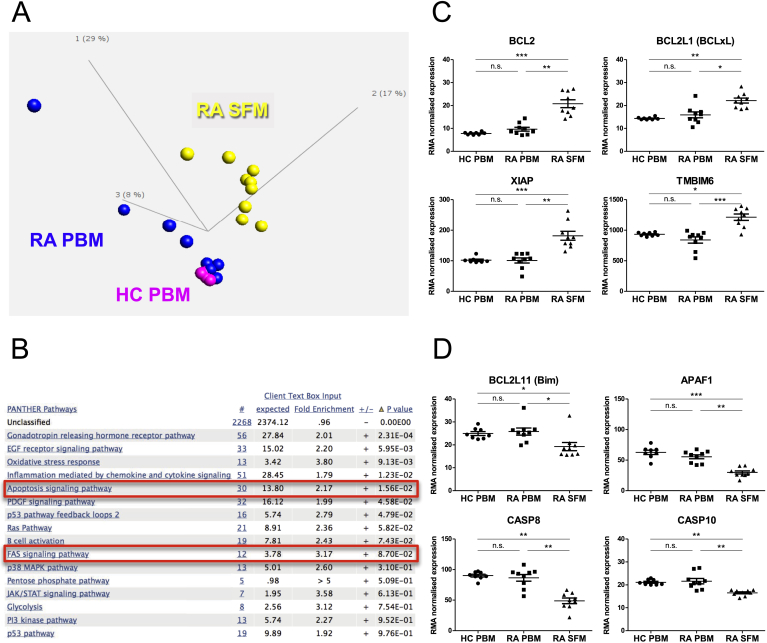
**Gene expression profiling shows decreased expression of pro-apoptotic genes in RA SFM**. (A) Principal component analysis plot of the three cell types assessed by gene expression profiling using Affymetrix arrays; healthy donor PB CD14+ cells (HC PBM, magenta), RA patient PB CD14+ cells (RA PBM, blue) and RA patient SF CD14+ cells (RA SFM, yellow). (B) Pathways that are over-represented in the 3033 differentially expressed genes (DEG, q < 0.05) in RA SFM vs. RA PBM. Pathway analysis was performed using the Panther database and p-values shown are after Bonferroni correction. The first numbered column (indicated by #) shows the number of genes from the DEG list that are classified in each pathway. (C, D) Array data showing (C) increased expression of pro-survival genes from the ‘apoptosis signalling pathway’ (highlighted in B) in RA SFM vs. PBM and (D) decreased expression of pro-apoptotic genes from the same set. (C) and (D) were tested by ANOVA, Kruskal-Wallis test with Dunn's post test. *p < 0.05, **p < 0.01, ***p < 0.001. (For interpretation of the references to colour in this figure legend, the reader is referred to the web version of this article.)

In order to understand possible changes in gene expression in the CD14+ cells from the site of inflammation compared to their circulating counterparts, an Affymetrix gene expression profiling study was undertaken examining nine SFM and PBM samples from patients with RA (of which n = 8 were paired) and eight PBM samples from age-matched healthy donors. No significant differences were observed between the profiles of RA and HC PBM, although there was considerable variation among the RA PBM samples. RA SFM however, formed a cluster distinct from both HC and RA PBM (Fig. 2A) and had 3033 significantly differentially expressed genes (DEG) relative to RA PBM (FDR 0.05) in an unpaired, two-group comparison. Pathway analysis of these DEG revealed that genes related to apoptosis signalling were statistically significantly over-represented in this set (Table 1 and Fig. 2B). Genes related to Fas signalling were also enriched, though not significantly. Among the 30 genes related to apoptosis signalling we found increased expression of the pro-survival genes *BCL2*, *BCL2L1* (Bcl-xL), *XIAP* and *TMBIM6* (Bax inhibitor) and decreased expression of the pro-apoptotic genes *BCL2L11* (Bim), *APAF1*, *CASP8* and *CASP10* (Fig. 2C and D). These data show that RA SFM have significant changes at the gene expression level, relative to PBM, that may contribute to the observed apoptosis resistance of these cells.

### 3.3 Mir-155 is highly expressed in RA SFM and may target pro-apoptotic transcripts

**Fig. 3:**
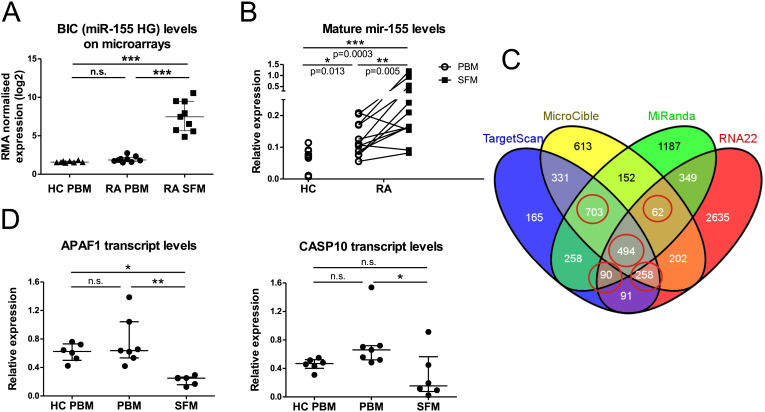
**Mir-155 is increased in RA CD14+ cells and may target apoptosis genes**. (A) Levels of the mir-155 precursor transcript BIC in HC PBM, and in RA PBM and RA SFM as assessed by gene expression profiling. (B) Levels of mature mir-155 were measured by TaqMan microRNA assay from total RNA isolated from FACS sorted PBM and SFM. Results were normalised to the small nucleolar RNA RNU48. HC PBM vs. RA PBM and HC PBM vs. RA SFM tested by Mann Whitney test; paired RA PBM vs SFM tested by Wilcoxon matched-pairs signed rank test. (C) Predicted targets of mir-155 from four software programs were overlapped and those predicted by all four and a combination of any three of the four were identified (circled). (D) Expression levels of the apoptosis-related genes APAF1 and CASP10 were measured by qRT-PCR in HC PBM (n = 6) and RA PBM (n = 7) and SFM samples (n = 6). Expression was normalised to the housekeeping gene SDHA. Multiple groups were tested by one-way ANOVA with Tukey's (A) or Dunn's post test (D). *p < 0.05, **p < 0.01, ***p < 0.001.

In the gene expression comparison of RA SFM vs. PBM, one of the most highly differentially expressed transcripts was *BIC* (B-cell Integration Cluster), which is the host gene for the microRNA mir-155, and which showed approximately 47-fold up-regulation in RA SFM vs. PBM (Fig. 3A). Mature mir-155 levels were also significantly increased in RA SFM relative to RA PBM as determined by qRT-PCR. Although there was no difference in host gene/*BIC* expression between RA and HC PBM, the qRT-PCR results showed significantly increased levels of mature mir-155 in RA PBM (Fig. 3B). Similarly, PsA SFM also showed significantly increased levels of mature mir-155 relative to HC and PsA PBM, however there was no difference in mature mir-155 levels between HC and PsA PBM (Supplementary Fig. 4).

As mir-155 is a well-studied miRNA in monocyte inflammation and since miRNAs function by regulating the expression of mRNA molecules, we sought to explore if there was a relationship between this miRNA and resistance to apoptosis in monocytes from RA patients. To identify potential mRNA targets of mir-155, we used predictions obtained from four different software programs (TargetScan, MiRanda, MicroCible and RNA22). Only those targets that were predicted by at least 3 of the 4 programs (Fig. 3C, circled) were included in further analysis. This list of predictions was then compared with the list of genes that were significantly down-regulated in the RA SFM vs. PBM microarray analysis, and that were apoptosis-related according to gene ontology analysis (Table 1). This analysis resulted in the identification of four candidate genes that are predicted targets of mir-155, are down-regulated in RA SFM and are known to be apoptosis-related. These genes were *APAF1, CASP10*, *FOS* and *PRKCB*. These genes were also found to be down-regulated in a recently reported independent gene expression profiling study examining the same cell types [32]. Of these four, *APAF1* and *CASP10* have been reported to be directly involved in apoptosis and decreased expression of their transcripts in RA SFM vs. RA PBM was confirmed by qRT-PCR in a set of independent samples (Fig. 3D). Together, these data suggest a potential direct role for mir-155 in monocyte/macrophage apoptosis through regulation of these two candidate transcripts.

### 3.4 Mir-155 over-expression increases survival and inflammatory potential of CD14+ cells

**Fig. 4:**
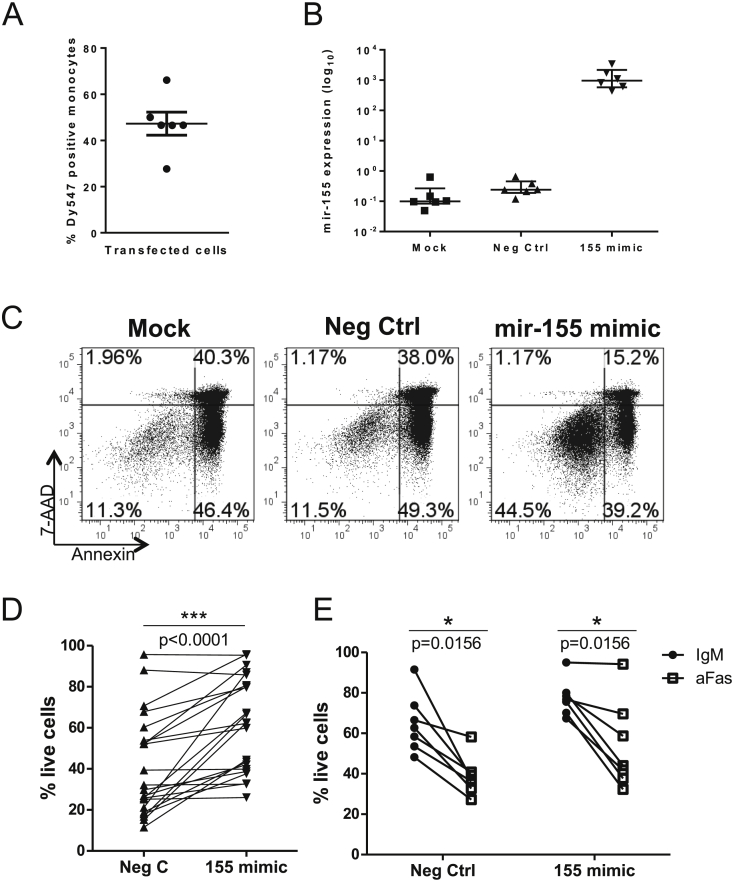
**Increased mir-155 promotes monocyte survival**. (A) Healthy donor monocytes were transfected with a negative control miRNA mimic conjugated to a fluorescent molecule Dy547. Following incubation for 40 h, Dy547 positive cells were assessed by flow cytometry. (B) Monocytes were transfected with mir-155 mimic or negative control mimic (Neg Ctrl), or with transfection reagent only (mock) and incubated for 40 h. Mature mir-155 levels were measured using a TaqMan microRNA assay. (C, D) Representative plots (C) and cumulative data (D, n = 22) showing healthy donor CD14+ monocyte survival 40 h after transfection with negative control mimic (Neg Ctrl) or mir-155 mimic. (E) Healthy donor monocytes were transfected (n = 7) with Neg Ctrl or mir-155 mimic for 24 h followed by overnight culture with an agonistic anti-Fas antibody (aFas) or the isotype control (IgM) at 200 ng/mL and the percentage of live cells assessed. D and E tested by Wilcoxon matched-pairs test.

To test the effect of increased mir-155 levels on CD14+ cell survival, miRNA mimics were used to overexpress either mir-155 or a non-targeting negative control in healthy PB CD14+ cells by transfection. Successful transfection was confirmed by flow cytometry of cells transfected with a fluorescent molecule (Dy547) conjugated mimic and showed that on average, 50% of the cells were transfected (Fig. 4A). Specific over-expression of mir-155 was confirmed by qRT-PCR in cells transfected with either the negative control, mir-155 mimic or mock transfected (Fig. 4B). Over-expression of mir-155 in healthy CD14+ monocytes significantly (17/22 experiments) increased the survival of these cells following 40 h culture, relative to control-transfected cells (Fig. 4C and D). A non-specific survival enhancing effect of the negative control mimic transfection was also observed (percentage of live cells following Mock 25% vs. Neg Control 41%, data not shown) however the cells transfected with mir-155 mimic had higher survival than either of these conditions (61%), indicating a specific, survival enhancing effect of mir-155. When mimic transfected cells were subjected to killing by the agonistic anti-Fas antibody (vs. control IgM) there was no survival benefit conferred by enhanced mir-155 expression (Fig. 4E).

**Fig. 5:**
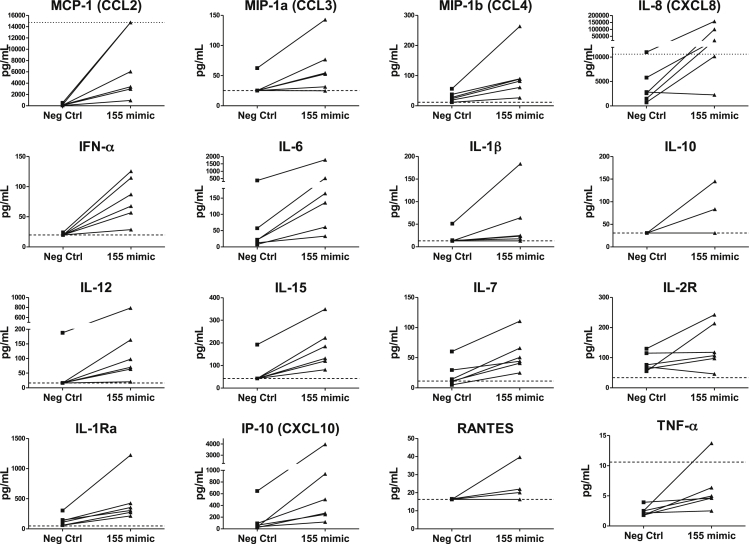
**Mir-155 over expression leads to increased monocyte cytokine/chemokine production.** Culture supernatants from healthy donor monocytes transfected with negative control (Neg Ctrl) or mir-155 mimic (as described in Fig. 4) were analysed using a Cytokine Human 25-plex Luminex assay; n = 6. Cytokines that were above the lower detection limit for at least 1 of the samples are shown. The dashed line shows the lower detection limit for the analyte, while the dotted line shows the upper detection limit.

In addition to increased survival, Luminex analysis revealed that over-expression of mir-155 in healthy CD14+ cells led to elevated expression of a range of chemokines and cytokines (Fig. 5). These included the chemoattractants MCP-1 (CCL2), MIP-1α (CCL3), MIP-1β (CCL4), IL-8 (CXCL8) and IP-10 (CXCL10) and the cytokines IFN-α, IL-6, IL-12, IL-15 and IL-7. IL-1Ra was also consistently increased. Soluble IL2R, IL-1β, TNF-α, IL-10 and RANTES were increased in some but not all samples, while the rest of the tested cytokines (IFN-γ, IL-4, IL-2, IL-17, Eotaxin, GM-CSF, IL-13, IL-5 and MIG, not shown) were not in the detectable range in any of the conditions.

## 4 Discussion

Monocytes and macrophages have a central role in the inflammatory processes in RA [1]. Here we propose that RA PBM and SFM may sustain this role through increased resistance to death and provide evidence that one of the factors that may assist in orchestrating this death resistance is the microRNA mir-155.

Two previous studies have reported on the resistance to spontaneous death of PBM of arthritic patients; the first examined PB CD33+CD3- cells from JIA patients in low-serum medium [20], while the second examined RA PBM in the presence of a control IgG antibody and low serum [19]. In our study, we tested highly pure preparations of RA PBM in 10% serum medium and no other additions.

Additionally, we demonstrate that RA SFM show enhanced resistance to spontaneous death relative to both HC and RA PBM, although this difference was lost when comparing PBM and SFM from the same patients in a small set of samples. The latter may be explained by enhanced apoptosis resistance in the PBM of these patients, which could potentially be due to a flare in disease activity. While this study was not specifically designed to assess any potential differences between PBM from patients who are or not undergoing a flare, the observation suggests that such acute inflammatory events may influence monocyte survival. In agreement with previous reports [16], [18], SFM were also found to be resistant to Fas-mediated death, induced by an agonistic anti-Fas antibody. The resistance to spontaneous and Fas-mediated death of SF-derived CD14+ cells may be a general feature of cells from the site of active inflammation in arthritis, since similar observations were made in PsA. Therefore, CD14+ cells from the inflamed joint appear to be resistant to death by both the extrinsic (death receptor) and intrinsic pathways of apoptosis.

One explanation for the observed relationship between increased monocyte survival and enhanced disease activity in RA is that systemic inflammatory factors may influence the susceptibility of monocytes to apoptosis induction. Alternatively, it is possible that monocytes/macrophages from patients with RA harbour intrinsic changes that drive both inflammation and resistance to apoptosis. Currently, it is not possible to discriminate between these two possibilities and both may occur in parallel.

In order to determine a possible molecular basis for the observed resistance to apoptosis, we undertook gene expression profiling to assess the differences between CD14+ cells from PB and SF. Although the profiling showed no statistically significant differences between PBM from RA patients and HC donors, as a whole, a notable feature of the gene expression profiling was the great degree of variation within the RA PBM samples (Fig. 2). Due to this variation in RA PBM but not HC PBM, we cannot definitively conclude that there is no difference between HC and RA PBM, or between subsets of RA patients. A repeat microarray experiment with a larger number of samples would be necessary to establish this. Interestingly, the variation within the RA PBM group is also reflected in the spontaneous apoptosis results (Fig. 1). These data also show large variation between patients' monocyte survival, with some of the RA PBM samples showing greatly increased survival compared to the majority of the HC PBM samples, whilst several other RA PBM samples clearly fall within the range observed in HC PBM.

The RA SFM gene expression profile however was clearly distinct. The differentially expressed genes in SFM (vs. PBM) were enriched for genes related to apoptosis signalling, an observation that supports our findings. While previous studies have reported increased expression of the proteins FLIP and MCL1 in RA SFM [16], [17], we found no significant differences in transcript levels of these molecules between the groups in our microarray data (not shown).

One of the most highly differentially expressed genes in RA SFM was the mir-155 host gene (also known as *BIC*). We show that the levels of mature mir-155 were also increased in RA SFM relative to RA PBM, supporting a previous report [31], and in RA PBM, relative to HC PBM, thus confirming a recent study [33]. The increase in mature mir-155 levels in SFM was also confirmed in PsA CD14+ cells (Supplementary Fig. 4). There was no difference in mir-155 levels between HC and PsA PBM, a finding that reflects the lack of difference in spontaneous monocyte survival between these two groups.

Mir-155 is highly conserved (present in all jawed vertebrates [34]) and is one of the best studied miRNAs in leukocytes. *BIC* and mature mir-155 were first reported as highly expressed in B-cell lymphomas [35] and have since been found to be involved in a range of cancers (reviewed in Ref. [36]). In cells of the monocyte/macrophage lineage, mir-155 is induced upon TLR ligation with various ligands [31], [37], [38] and is involved in both positive and negative regulation of inflammation through mechanisms including repression of the immune modulators SHIP-1 [25], SOCS1 [39] and BCL6 [40].

Dysregulation of mir-155 has been observed in human autoimmunity, with increased levels reported in active ulcerative colitis (vs. HC) [41] and in multiple sclerosis CNS tissue [42]. In RA, increased expression of mir-155 has been reported in PBMC, synovial fibroblasts and synovial tissue [26], [27], [30] and in RA SFM, in which it represses SHIP-1 [31]. In addition, mir-155 (whole-organism) knockout mice were found to be resistant to CIA [31], [43]. Our findings in this study of increased mir-155 levels in RA SFM and PBM and a pro-survival and pro-inflammatory role are therefore consistent with existing literature. We can also confirm a decrease in *INPP5D* (encoding SHIP-1) levels in RA SFM corresponding with an increase in mir-155 levels in these cells (Supplementary Fig. 5).

One of the major bottlenecks in miRNA target discovery is the sheer number of mRNAs that are bioinformatically predicted for each miR, and the relatively low level of overlap between the predictions of each program. To circumvent these issues we chose to consider predictions made by at least three of four programs, thus identifying four candidates that are apoptosis related and are down-regulated in RA SFM in microarrays done by us as well as microarrays performed independently by another group [32]. Down-regulation of two candidate genes, *APAF1* and *CASP10*, in RA SFM was confirmed by q-RT-PCR. The genes *APAF1* and *CASP10* are both known players in apoptotic processes and represent the two apoptosis pathways: the extrinsic or death receptor mediated pathway (CASP10) and the intrinsic or mitochondrial pathway (APAF1).

Mimicking the mir-155 over-expression seen in RA SFM by transfecting HC PBM to over-express this miR resulted in significantly increased survival of these cells. A direct effect of mir-155 on spontaneous cell survival has not previously been shown. In contrast, mir-155 overexpression did not enhance survival of monocytes upon addition of the agonistic anti-Fas antibody, suggesting that the effect of mir-155 may be restricted to the intrinsic pathway and APAF1.

Further investigation by PCR of expression of *APAF1* in cells from mimic transfected wells did not show a consistent decrease in transcript levels after mir-155 mimic transfection (Supplementary Fig. 6). However, this may not be surprising; a lack of effect of mir-155 on *APAF1* transcript, but an observable effect on protein level, has been reported previously in a study that confirms APAF1 as a target of mir-155, albeit in a different context [44]. We did not observe a consistent effect on *CASP10* transcript, but did note a decrease in *INPP5D* expression in mir-155 mimic transfected monocytes at 24 hours. This difference was not seen at 40 hours, the time point at which monocyte survival was assessed. This indicates the possibility of other factors coming into effect at this later time point rendering it difficult to measure the effect on the transcript. The *INPP5D* transcript has been extensively biologically validated as a target of mir-155 and therefore the lack of a clear relationship between its expression and mir-155 overexpression suggests that the chosen method may not be ideal for measuring this effect. The observed variation could be due to the fact that only part of the cells (avg. 50%) were transfected with the mimic while we assessed transcript levels in all the cells in the well. As these were primary cells, when plated they may be at different stages of their life cycle, and therefore have had different amounts of target transcript levels to begin with.

The other significant outcome of mir-155 over-expression was pro-inflammatory cytokine production. Previous studies have shown increased TNF-α in human monocyte-derived macrophages [42] and increased TNF-α, IL-6, IL–8 and IL-1β in RA PBM [31] following mir-155 over-expression. We extend these data by showing that several other cytokines and chemokines (MCP-1, MIP-1α, MIP-1β, IL-8, and IP-10, IFN-α, IL-6, IL-12, IL-15 and IL-7) are also upregulated by overexpression of this miR. The changes in cytokine levels we detected were a direct consequence of mir-155 over-expression, as no other stimulus was provided to the cells. These findings are supported by a recent study that reported a mir-155 mediated increase in the receptors for many of these chemokines [33]. Although we cannot rule out the possibility that the increase in detected cytokines could be due to increased numbers of surviving cells, rather than increased production per cell, the resulting wide-ranging influence of the induced mediators suggests a role for mir-155 in monocyte/macrophage-driven inflammatory processes in RA. For instance, increased SFM production of MIP-1α, MIP-1β, MCP-1 and IL-8 and increased IL-12, IL-15, IL-7 would recruit myeloid and lymphoid cells to the joint, respectively.

## 5 Conclusions

Collectively, our data show increased resistance to cell death in RA SFM and PBM and a concurrent increase in the expression of mir-155. We also demonstrate that increased mir-155 expression in healthy CD14+ cells renders them more resistant to death and enhances their cytokine/chemokine production These findings suggest that the increased expression of mir-155 in PBM and SFM of patients with RA may contribute to inflammation through two means: first, by increasing monocyte/macrophage survival, possibly by directly repressing expression of pro-apoptotic genes; and second, by increasing levels of a wide range of pro-inflammatory cytokines and chemokines.

## Declaration of potential conflict of interest

This study was supported by a research grant from Novo Nordisk A/S. CBR and KSF were employees of Novo Nordisk A/S.

## Figures and Tables

**Table 1:** **Genes that are significantly differentially expressed in RA SFM (vs. RA PBM) and are classified as related to ‘apoptosis signalling’ by Panther gene ontology database.** Gene ontology and pathway analysis was performed on the 3033 differentially expressed genes between RA SFM and PBM using the Panther database (www.pantherdb.org). Using this tool a statistical overrepresentation test was performed and the resulting panther pathways categories after a Bonferroni analysis for multiple testing are shown in Fig. 2B. The genes in the category ‘apoptosis signalling’ are shown in this table, separated by those increased in SFM vs. PBM and those that are decreased.

Gene symbol	Gene name
**Increased in SFM vs. PBM**	
HSPA1A	Heat shock 70 kDa protein 1A
BCL2L1	BCL2-like 1 (BCL-XL/S)
BAG3	BCL2-associated athanogene 3
MAPK7	Mitogen-activated protein kinase 7 (ERK5)
HSPA6	Heat shock 70 kDa protein 6 (HSP70B)
MAPK8	Mitogen-activated protein kinase 8 (JNK1)
HSPA2	Heat shock 70 kDa protein 2
BCL2	B-cell CLL/lymphoma 2
TNFRSF10D	TNF Receptor Superfamily, Member 10d, decoy with Truncated Death Domain (TRAIL4)
XIAP	X-linked inhibitor of apoptosis
MAP4K3	Mitogen activated protein kinase kinase kinase kinase 3
CASP7	Caspase-7, apoptosis-related cysteine peptidase
TMBIM6	Transmembrane BAX inhibitor motif containing 6
ATF2	Activating transcription factor 2 (CREB2)
HSPA5	Heat shock 70 kDa protein 5 (glucose regulated protein 78 kDa)
PIK3CB	Phosphatidylinositol-4,5-bisphosphate 3-kinase, catalytic subunit beta
**Decreased in SFM vs. PBM**	
HSPA1L	Heat shock 70 kDa protein 1-like
LTB	Lymphotoxin beta (TNF superfamily, member 3) (TNFSF3)
PRKCB	Protein kinase C, beta
MAP4K2	mitogen-activated protein kinase kinase kinase kinase 2
FOS	FBJ murine osteosarcoma viral oncogene homolog
CASP10	caspase 10, apoptosis-related cysteine peptidase (ALPS2, FLICE2)
CASP8	caspase 8, apoptosis-related cysteine peptidase (ALPS2B, FLICE)
BCL2L11	BCL2-Like 11 (Apoptosis Facilitator) (BIM)
APAF1	Apoptotic Peptidase Activating Factor 1
MAP3K5	Mitogen-activated protein kinase kinase kinase 5
BAG4	BCL2-associated athanogene 4
PIK3CD	Phosphatidylinositol-4,5-bisphosphate 3-kinase, catalytic subunit delta
TP53	Tumor protein p53
TNFRSF10C	Tumor Necrosis Factor Receptor Superfamily, Member 10c, decoy without an intracellular domain
