# Supplementary material for: MicroRNA-155 contributes to enhanced resistance to apoptosis in monocytes from patients with rheumatoid arthritis
Source: J Autoimmun. 2017 May;79:53–62. doi: 10.1016/j.jaut.2017.01.002 (PMC5397583; doi:10.1016/j.jaut.2017.01.002)
Supplement: Supplementary file 1 [file mmc1.pdf]

Supplementary Table 1

	Microarrays			Apoptosis assays		
	**RA PBM (n=9)**	**RA SFM (n=9)**	**HC PBM (n=8)**	**RA PBM (n=21)**	**RA SFM (n=8)**	**HC PBM (n=16)**
**Female / Male**	7/2	8/1	6/2	4/17	1/6, 1 n/a	12/4
**Age in years (mean±SD)**	60.1 (± 17.1)	62.1 (± 16.6)	50.9 (± 11.8)	52.7 (±15.1) (n=19)	46.9 (±18.8) (n=7)	38.8 (±12.3) (n=16)
**DAS28 score (mean±SD)**	4.9 (± 1.3)	4.8 (± 1.2)	-	4.5 (± 1.3) (n=20)	4.7 (± 1.4) (n=7)	-
**ESR [mm/hr] (mean±SD)**	31.4 (±22.0) (n=8)	30.8 (± 22.0) (n=8)	-	26.0 (± 21.6) (n=18)	46.0 (± 25.7) (n=7)	-
**CRP [mg/l] (mean±SD)**	20.3 (± 19.4)	22.3 (± 18.7)	-	13.5 (± 13.0) (n=15)	18.75 (± 13.3) (n=4)	-
**Treatment (None/ DMARD/ Biologic)**	3/ 4/ 2	4/ 4/ 1	-	3/ 16/ 2 (n=21)	2/ 4/ 1 (n=7)	-
**Rheumatoid Factor (+/-)**	8/1	8/1	-	13/7 (n=20)	3/2 (n=5)	-
**Erosive (Y/N)**	7/2	6/3	-	9/9 (n=18)	4/1 (n=5)	-
**Disease duration**	19.7 (±19.0) (n=6)	17.57 (±18.3) (n=7)	-	9.6 (±12.4) (n=17)	14.3 (±13.2) (n=7)	-

Supplementary Table 1. Demographic and clinical parameters of the patients and healthy donors whose samples were included in the study. Among the microarray samples, 8/9 PBM and SFM were paired. Among the samples used for apoptosis assays 6/8 SFM samples had paired PBM samples. Abbreviations used: CRP, C-reactive protein; DAS28, disease activity score of 28 joints; DMARDs, disease-modifying anti-rheumatic drugs; ESR, erythrocyte sedimentation rate.

Supplementary Figure 1

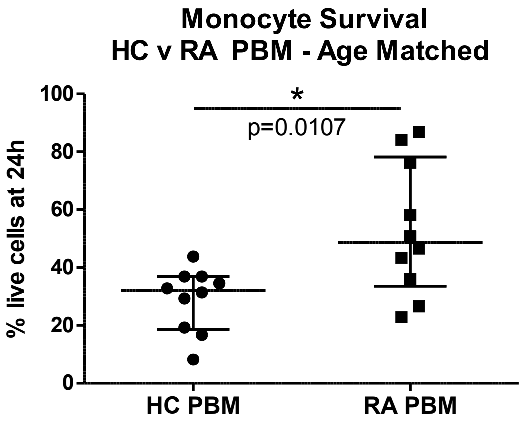

Supplementary Figure 1. Enhanced monocyte survival in RA PBM. CD14+ monocytes were cultured for 24 hours and the percentage of surviving monocytes determined as described in Figure 1. HC and RA PBM survival was compared in age-matched samples (mean age ± SEM in HC 43.0 ± 4.3 years, in RA 43.2 ± 4.2 years). Graphs show medians with inter-quartile range. Data were normally distributed and tested by unpaired t-test with Welch's correction.

Supplementary Figure 2

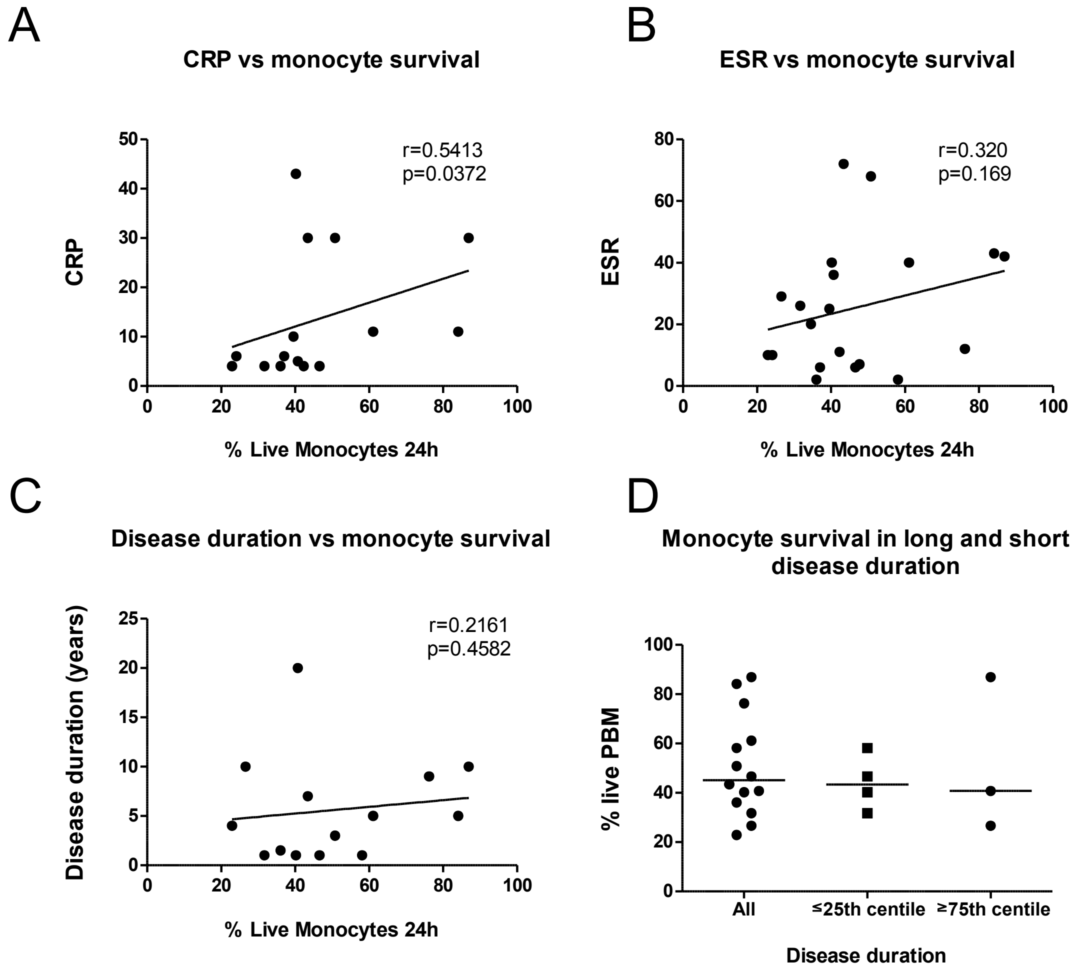

Supplementary Figure 2. Correlation of monocyte survival with disease parameters. Peripheral blood CD14+ monocytes from patients with RA were cultured for 24 hours and the percentage of surviving monocytes determined as described in Figure 1. Patients' (A) CRP (C-reactive protein) (B) ESR (erythrocyte sedimentation rate), and (C) disease duration were correlated with monocyte survival. The correlations were tested by Spearman's R. (D) Monocyte survival was compared in samples from the subset of patients from (C) that had disease duration in the 25th or lower percentile or in the 75th or higher percentile of all patients tested in our study. Data were analysed by a one-way ANOIVA; no significant differences were observed.

Supplementary Figure 3

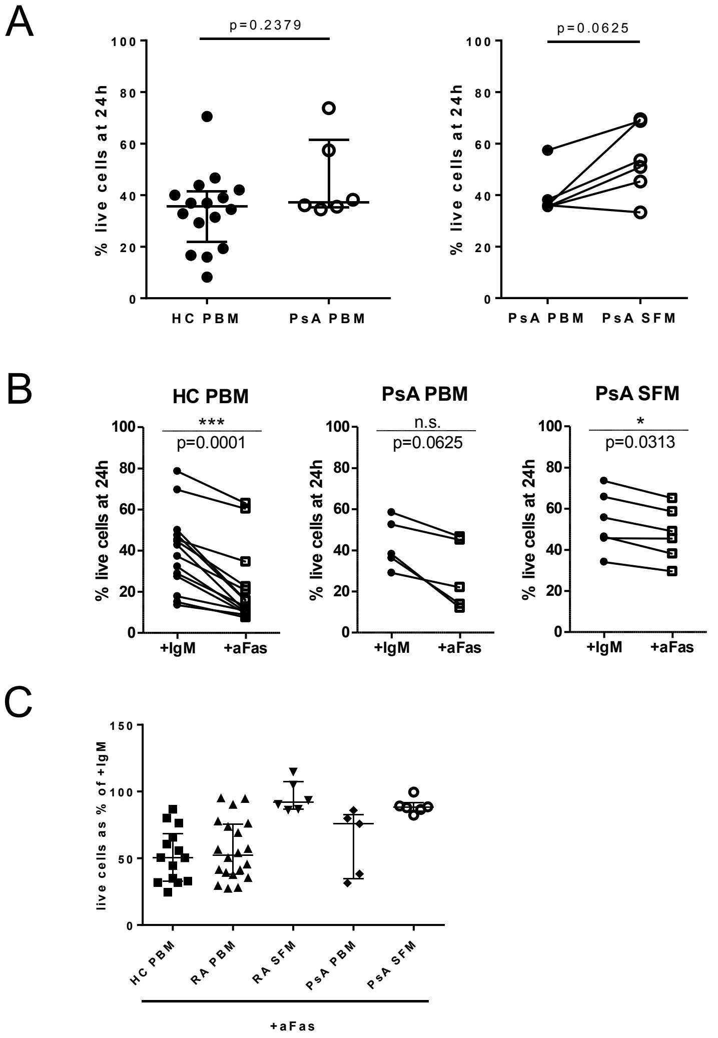

Supplementary Figure 3. Monocyte survival in PsA. (A) CD14+ monocytes from peripheral blood and synovial fluid from patients with PsA were cultured for 24 hours and the percentage of surviving monocytes determined as described in Figure 1. Graphs show cell survival at 24 hours by HC PBM (n=16) vs PsA PBM (n=6) (left graph, median with inter-quartile range, Mann-Whitney test) or by paired PsA PBM vs SFM (n=4, with two patients having SF in both left and right knee) (right graph, Wilcoxon matched-pairs signed rank test). (B, C) HC PBM (n=14), PsA PBM (n=5) and PsA SFM (n=6) were cultured with isotype control or with an agonistic anti-Fas antibody as described in Figure 1. Data shown as percentage live cells after treatment with isotype control vs. anti-Fas antibody (B) or plotted relative to control IgM antibody (C). Data were tested by Wilcoxon matched-pairs signed rank test. *p<0.05, ***p<0.001.

Supplementary Figure 4

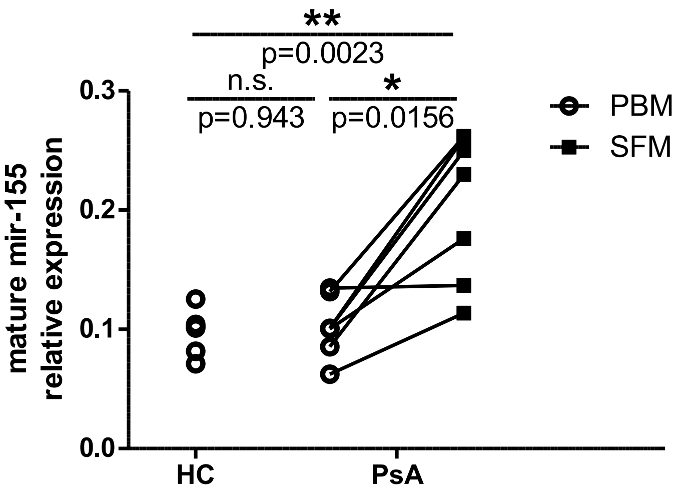

Supplementary Figure 4. Mature mir-155 levels in PsA CD14+ cells. Levels of mature mir-155 in HC and PsA PBM and PsA SFM measured by qPCR. PsA PBM vs SFM tested by Wilcoxon matched-pairs signed rank test, the rest by Mann-Whitney test.

Supplementary Figure 5

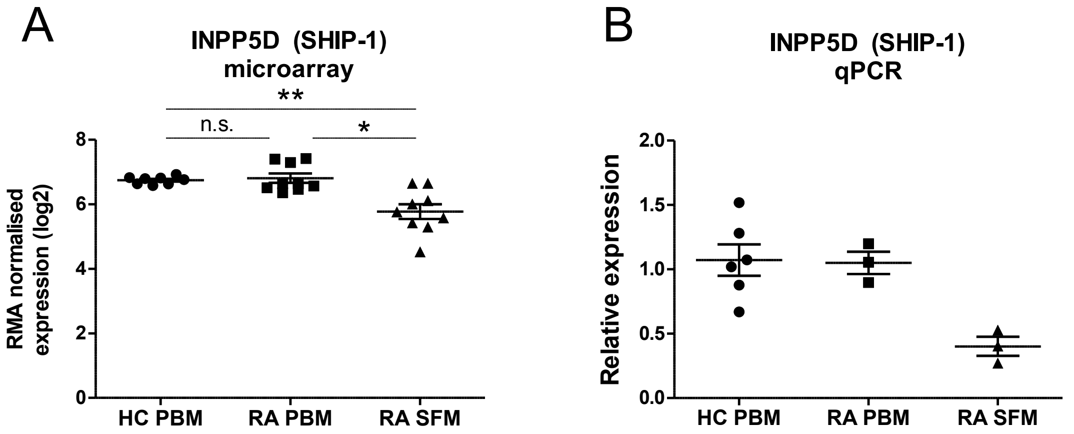

Supplementary Figure 5. Expression of *INPP5D* transcript in monocytes from healthy donors and RA patients. Expression of *INPP5D* determined by Affymetrix arrays as described in Figure 2, and (B) confirmed in HC PBM (n=6) and paired RA PBM and SFM (n=3) by q-RT-PCR. Groups were tested by one-way ANOVA (Kruskal Wallis test) with Dunn's post test.

Supplementary Figure 6

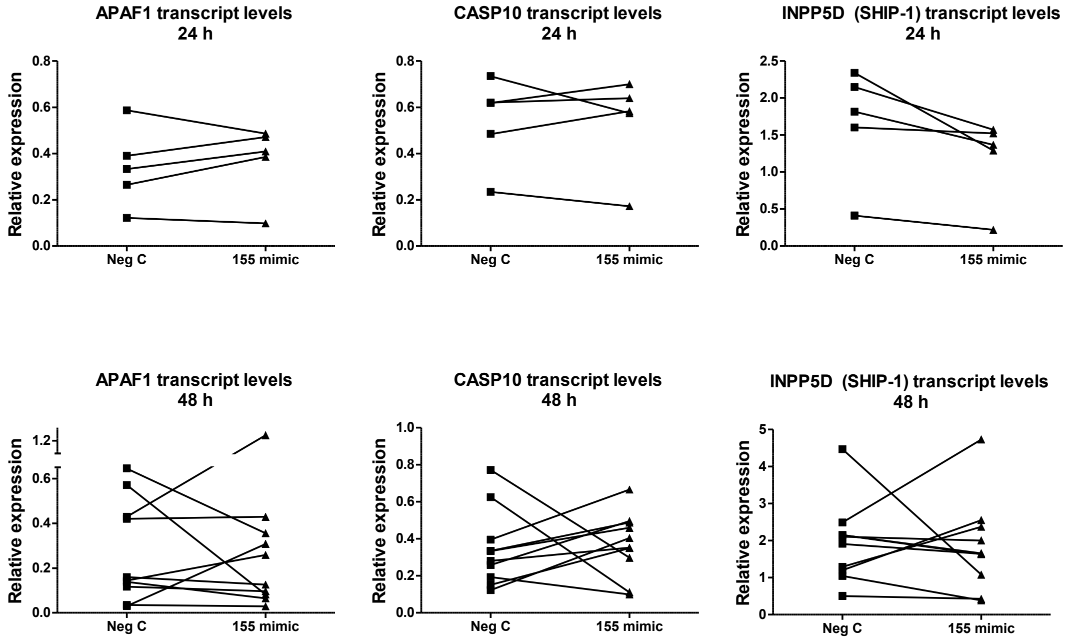

Supplementary Figure 6. Expression of *APAF1*, *CASP10* and *INPP5D* transcript in monocytes from healthy donors transfected with control or mir-155 mimic. Peripheral blood CD14+ cells from healthy donors were transfected with negative control or mir-155 mimic. All were cells collected for RNA at 24 (top panel, n=5) or 48 (bottom panel n=10) hours after transfection and transcript levels quantified by q-RT-PCR. Transcript level was normalised to the housekeeping gene *SDHA*.
